# A Multi-Scale–Multi-Stable Model for the Rhodopsin Photocycle

**DOI:** 10.3390/molecules190914961

**Published:** 2014-09-18

**Authors:** Francesco Tavanti, Valentina Tozzini

**Affiliations:** 1 NEST-Istituto Nanoscienze, CNR, Piazza San Silvestro 12, 56127 Pisa, Italy; 2 Department of Physics “E. Fermi”, University of Pisa, Largo B. Pontecorvo 3, 56127 Pisa, Italy; 3 Scuola Normale Superiore, Piazza San Silvestro 12, 56127 Pisa, Italy

**Keywords:** rhodopsin, multi-scale modeling, classical molecular dynamics, coarse grained models, computer simulations

## Abstract

We report a multi-scale simulation study of the photocycle of the rhodopsins. The quasi-atomistic representation (“united atoms” UA) of retinal is combined with a minimalist coarse grained (CG, one-bead-per amino acid) representation of the protein, in a hybrid UA/CG approach, which is the homolog of QM/MM, but at lower resolution. An accurate multi-stable parameterization of the model allows simulating each state and transition among them, and the combination of different scale representation allows addressing the entire photocycle. We test the model on bacterial rhodopsin, for which more experimental data are available, and then also report results for mammalian rhodopsins. In particular, the analysis of simulations reveals the spontaneous appearance of meta-stable states in quantitative agreement with experimental data.

## 1. Introduction

The rhodopsins are seven helix transmembrane photoactive proteins, whose function is triggered by the adsorption of a photon in the visible region by mean of the co-factor retinal included in the active site of an opsin [1,2]. This study focuses on the two main representatives of this group, namely the bacterial rhodopsin (BR) produced by *Halobacterium salinarum*, a prokaryotic rhodopsin, whose function is to pump protons from the intracellular to the extracellular side and maintain an electrochemical gradient [3], and the mammalian rhodopsin (MR), an eukaryotic rhodopsin, present in the membrane of the photoreceptor cells of the retina and responsible of the first step of the vision process (*i.e.*, the photon absorption [4]). 

**Figure 1:**
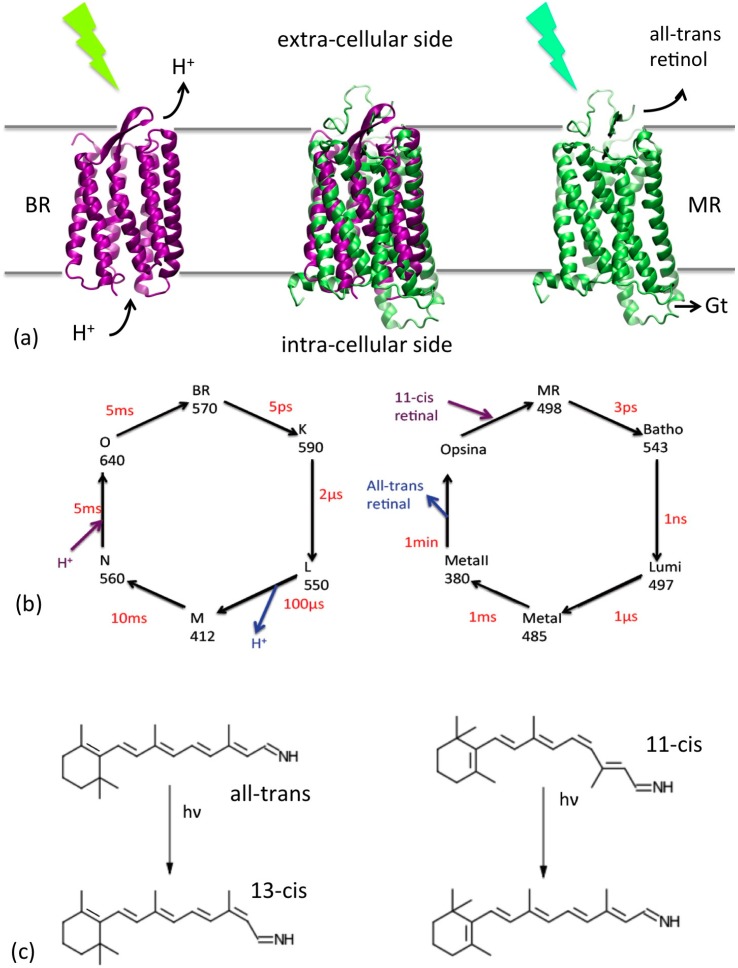
(**a**) Cartoon representations of the BR (purple) and MR (green) and their location with respect to the cell membrane. Their superposition is reported in the center. Their functions (triggered by a photon absorption) are also indicated: proton translocation for BR and interaction with the G-protein transducine (Gt) for MR, which initiate the cascade of events of the vision process. (**b**) Schematic representation of the photocycles of the two proteins. The conventional name of each state, their adsorption wavelengths and their average life time are reported. (**c**) Retinal photoisomerizations for BR (left) and MR (right).

Despite their different function, the two proteins share a common operation mechanism (see Figure 1): the photon is absorbed by the retinal located in the active site, which undergoes a *cis*-trans (or *trans-cis*, see Figure 1c) isomerization [5] of one of the C=C bonds in the tail; this triggers a number of structural transitions resulting in the proton translocation in the case of BR [6,7] and in the expulsion of the retinal and interaction with transducing G protein, in the case of MR [8], which in turn produces a cascade of events generating the visual nerve impulse. Both the cycles involve six different structural states with different optical properties (Figure 1b), connected by barriers of different heights, up to several tens of Kcal/mole [9,10], implying kinetics ranging from the fs scale (*i.e.*, the photoinduced retinal isomerization) to the msec for BR or min for MR. 

Given the large range of different time scales involved in the process, it is clear that the simulation approach to this system cannot be based on single methods: to represent the fast structural transitions accurate atomistic representations are necessary, but these cannot reach the macroscopic time scale in the simulations. Thus, the rhodopsins have always been a privileged test case for multi-scale molecular dynamics simulations. Since the first step of the photocycle is a *cis-trans* isomerization following a photoexcitation of the retinal, several studies have mixed quantum representation (QM) of the retinal (and possibly active site) and classical molecular dynamics [11] (MD) empirical force field (FF) based simulations for the opsin, in the so-called hybrid QM/MM approach [12,13]. In those cases, however, the bottleneck of the calculation is the QM part, which limits the total simulation length to the scale of ps, excluding a relevant part of the photocycle. 

**Figure 2:**
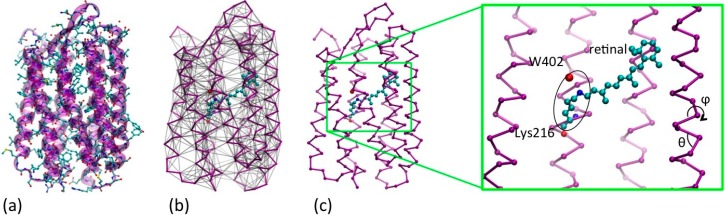
(**a**) Quasi-atomistic representation of the BR superimposed to cartoon representation (**b**) UA/CG representation of the protein: opsin minimalist model in purple and the retinal and water402 in quasi atomistic representation, colored by name (C = cyan, N = blue, O = red). The network of non-bonded interactions is represented in grey (**c**) The same as (b), without the non-bonded interactions for clarity, and with an expaned view of the active site. The retinal, the Schiff base linkage with Lys216 and the water are highlighted, as far as the internal conformational variables of the CG opsin model (θ,ϕ).

In order to reach larger time scales, in this work we scale up in the multi-scale representation, and mix a quasi-atomistic (United Atoms‒UA [14]) representation of the active site with a coarse grained (CG) minimalist (one-bead per amino-acid) representation [15] of the opsin (see Figure 2) in a UA/CG approach [16]. Our approach is classical, the units (the heavy atoms and the CG “beads”) interacting with empirical FF. This ensures a the possibility of addressing in simulations both the fast triggering cis-trans transition of the retinal and the slow subsequent conformational transitions, giving a global view of the whole photocycle. However, the coherency among the different resolution levels and the accuracy of the representation is here achieved by an accurate choice of the functional forms of the FF terms and by combining two different kinds of parameterization strategies: inclusion of information from different experimental sources (structural and energetic data, the “top down” parameterization) and from higher resolution representation (“bottom up”). For this reason, the QM level, though explicitly absent, is implied in the parameterization of the retinal FF.

This paper is organized as follows: in the next section we illustrate our multi-scale approach and the parameterization strategies. This is already an original part of this work, since the multi-scaling and parameterization procedures are not standard. We then report and discuss results on BR, which is also considered as a test case, given the large number of structural data available for this system, and on the MR (specifically the bovine rhodopsin). Conclusion and perspectives follow, and are followed by a brief Experimental Section including all the standard parts of the methods and detailed simulation settings.

## 2. UA/CG Models of Rhodopsins

### 2.1. Model and Force Field Definition

The multi-scale modeling procedure here reported is illustrated for the BR in Figure 2 (and then repeated analogously for MR). The structure is represented at two levels of resolution. The “active site”, which we define composed by the retinal, the side chain of Lys216 (and their Schiff linkage), and the water involved in the molecular mechanism of the protein (e.g., W402 for BR, see Figure 2c), are represented at a quasi-atomistic level: an interacting center (“bead”) represent a “united atom” [17], namely an “heavy atom” (C, O or N) and its saturating hydrogens (ball & sticks representation in color in Figure 2). Conversely, the opsin part (seven helices plus connecting loops) has a resolution of a single bead per amino-acid, with the bead located on the Cα (in purple in Figure 2), as in the “minimalist models” previously used for a number of different proteins [18]. The solvent and the membrane are implicitly treated, thus the model for BR has 253 interacting centers in total, approximately two orders of magnitude less than an atomistic model in explicit solvent.

The FF is formally separated in three parts:

$$U=U_{CG-CG}+U_{UA-UA}+U_{UA-CG}$$

representing the intra-opsin, intra-active site, and opsin-active site interactions, respectively. These all share a common separation in FF terms:

$$U_{XX-YY}=U_{l}+U_{\theta }+U_{\phi }+U_{nb}$$

the first three “bonding” terms representing the (pseudo)bond, (pseudo)bond angle and (pseudo)dihedral terms respectively, while the last one represent all the physically non bonded interactions (electrostatics, hydrophobicity, hydrogen bonding, excluded volume and internal flexibility effects). This is conveniently separated in two parts:

$$U_{nb}=U_{loc}+U_{non-loc}$$

*U_loc_* including the stronger and more directional part of the interactions, and *U_non-loc_* the remaining, usually more isotropic, ones. The local-non-local separation, as far as the topology of the bonded part and the functional forms of the different terms depend on the class of interaction (CG-CG, CG-UA, UA-UA), as described in the following.

The intra-opsin (CG-CG) FF is inspired by the minimalist models for proteins: the three bonding terms are particularly simplified by the linear chain topology, and represented by sums of two, three, and four beads terms, respectively [19], depending on the distance, bond angle and dihedral, with functional form depending on the secondary structure [20]. The non-bonded interactions are sums of pair-wise Morse-like potentials. The separation between bonded and non-bonded interactions is based on a purely geometrical criterion (*i.e.*, cutoff radius), the local part retaining a bias towards a reference structure while the non-local describing a generic-unbiased amino-acid interaction. Details of the functional forms and parameters are given in the Supplementary Information, Section S3. This scheme has proven an efficient compromise between simplicity and flexibility, allowing the simulation of complex motion of a large number of bio-systems of different size and flexibility [21,22]. In this work, the flexibility of the model is further enhanced, by allowing transitions between different states as described in Section 2.3.

The rest of the FF for the active site is built similarly, with variants due to the different physico-chemical nature of the beads. For the active site part, these are branched bonding topology, atom type dependent functional forms of *U_non-loc_*. The starting parameterization for the FF is taken from standard UA models [17] and then optimized as specified in Section 2.2 and Section 2.3 (complete set of functional forms and parameters given in the Supplementary Information, Section S3). The UA/CG interface interactions between the active site and the opsin includes a few bonded interactions describing the linkage between Lys216 side chain and the backbone and non-bonded local and non-local interactions. The latter include also the hydrogen bonds formed by the free waters with amino acids of the active sites, which are treated by specific Morse terms. The bonded terms and *U_local_* are parameterized and optimized with the same procedure as the active site, while for the parameterization of the non local terms and of hydrogen bonds formed by the free waters we followed a specific optimization procedure based on atomistic simulations, which is described in the next section.

The interaction with the solvent is implicit in the non-bonded interactions. The membrane constraining effect is simulated with a tethering potential applied to the opsin beads exposed to the membrane in the form:

$$U_{teth}=\frac{1}{2}{\text{K}}_{teth}\text{Δ}r^{2}$$

with Δ*r* the displacement of the bead with respect to its starting position. *K*_teth_ is optimized as describe in the following section and given in the Supplementary Information (Section S3) together with all the parameters of each of the FF term.

### 2.2. Single States Multi-Scale Model Building and Parameterization

Both BR and MR include six different states within the photocycle, optically distinguishable and structurally different (see Figure 1). The main structural differences between the states involve the retinal structure and the turns regions, and some global helical movements in specific stages of the cycle (see the Supplementary Information, Section S2 for details) [23,24,25,26]. The parameterization of the multi-scale models requires reference structures for each of the states, on which the parameterization of the bonding and local terms is based. In the case of BR these are available in the PDB database [27]. When multiple structures were present, the reference one was chosen on the basis of the resolution and/or completeness. Incomplete structures were completed by homology. This procedure and the final choice of the states are described in the Supplementary Information. 

MR is larger than BR by about 100 amino-acids, mainly distributed in the loops connecting the helices and in a short transversal intra-cellular helix in the C terminal portion. As for BR, the retinal is covalently connected to a lysine side chain (Lys296 in this case), and the retinal occupies the central region of the helix bundle, with slightly different relative orientation. Interestingly, the rest states of the two proteins (BR for bacteriorhodopsin and MR for mammalian rhodopsin see Figure 1) have the retinal in different configurations (all-*trans* for BR and 11-*cis* for MR). Experimental determinations of the structural states of MR (specifically of bovine rhodopsin) are available only for the rest state (MR) and for the LUMI state (see the Supplementary Information Section S2 for details). In addition, the seven helices’ organization is sensibly different in the two cases, which hinders the homology modeling of MR based on BR. Thus for bovine rhodopsin we focus on MR and LUMI states. 

The parameters are taken from previously optimized models, when possible. As said, the opsin CG parameterization is inspired by the minimalist models for proteins of [20,22], but optimized with respect to these: the secondary structure dependence of the bond angle elastic constants is made more accurate and is also introduced in the dihedral term; the functional dependence of the energetic parameters of the non-bonded interactions was optimized (with negligible change with respect to previous parameterization, see Supplementary Information, Section S3 for details and numerical values). The parameter optimization (included the *K*_teth_) is performed by comparison with root mean squared fluctuations (RSMF, see also Experimental Section) either experimental or taken from *ad hoc* performed atomistic simulations with OPLS FF [28] (see the Experimental Section). 

The parameterization of the active site UA model combines the united atoms FF parameterization available in [14] with the reference structure based philosophy, to make the model compatible with the opsin part: the reference structures are used for the equilibrium parameters and the UA FF for the elastic constants. For the non-bonded interactions, the Lennard Jones parameters of [14] are used.

The UA/CG interface interactions needed a *de novo* parameterization, which however was performed the same philosophy of the others, to preserve coherency within the model. The elastic constants of the linkage between the retinal-Schiff base with the opsin are taken from the UA parameterization [14]. The non-bonded interactions occurring at the interface (e.g., the hydrogen bonds between the active site water and the opsin residues) were represented with Morse interactions, and the parameters fitted on energy profiles evaluated with atomistic calculations (see Supplementary Information Section S3 for the details of the procedure and for the optimized parameters). 

The parameters set build with this procedure for BR coherently combines UA and CG previously existing parameterizations with minimal adjustments, and it is used with no change for MR, except for the different reference states.

### 2.3. Multi-Stable Model Building

Our aim here is not only to simulate the intrinsic dynamics of the single states, but also the transitions between states. To this purpose, we built multi-stable FFs by coupling subsequent states. The connecting FFs are built with a procedure that is a variant of the plastic and double well network models [29,30,31]. In particular, for each pair of reference states A and B we select within the FF a subset of single interactions *u*(*x*) (where *x* can be a single bond angle, dihedral or non-bonded interaction) which changes most between the two states. These pairs of homologous interactions *u^A^*(*x*) and *u^B^*(*x*) are substituted by:

$$u^{A}(x)=\frac{\left[u^{A}(x)+\left(u^{B}(x)-\delta \right)\right]-\sqrt{{\left[u^{A}(x)-\left(u^{B}(x)-\delta \right)\right]}^{2}+\varepsilon^{2}}}{2}$$

obtaining a potential interpolating between the two parent ones. The operation is repeated for all the potentials terms in the subset (except those whose variation between states is negligible, see Supplementary Information Section S3 for details), leading to a FF smoothly interpolating between the two states. The parameter ε is small and serves uniquely to avoid the formation of a cusp between the two wells. The parameter δ, conversely, determines the relative stability of the two states. In order not to introduce additional adjustable parameters into the model, we assigned δ based on the free energy difference between states A and B, which is known experimentally for each transition, (see Supplementary Information Section S5 for the numerical values and for other details about the double well functional forms). The inclusion of macroscopic thermostatistic data, and, in general the mixing of bottom-up with top-down approaches, has proven an efficient strategy to obtain accurate parameterization for models at different levels of coarse graining [15,20,21,22,32].

We remark that no additional parameters are included to control the barrier heights. These were however evaluated subsequently in the photocycle dynamics simulations and additionally estimated along approximated reaction paths (see Results section). 

## 3. Results and Discussion

### 3.1. Single States Dynamics

**Figure 3:**
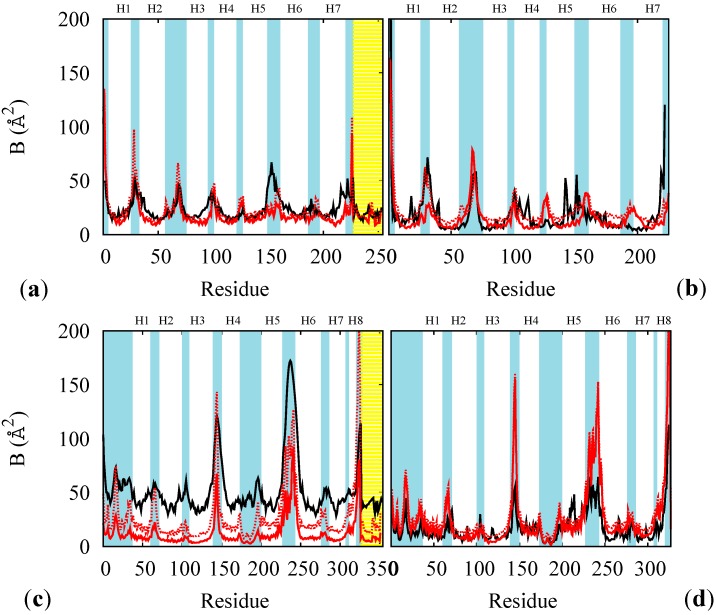
B factors of the rest states of BR (**a**,**b**), and of MR (**c**,**d**), at 100 K for the whole proteins (a,c) and at 300 K for opsin only (b,d). Red lines: results from multi-scale simulations with (solid) and without (dotted) containing potential for the membrane. Black lines: experimental data at 100 K ((a) PDB code 1M0K and (c) PDB code 1U19) or from atomistic thermalized simulations of hydrated opsin at 300 K (of BR (b) and of MR (d)). The yellow shade indicates the retinal and the cyan shades the loops regions. The helices are labeled as Hn.

**Figure 4:**
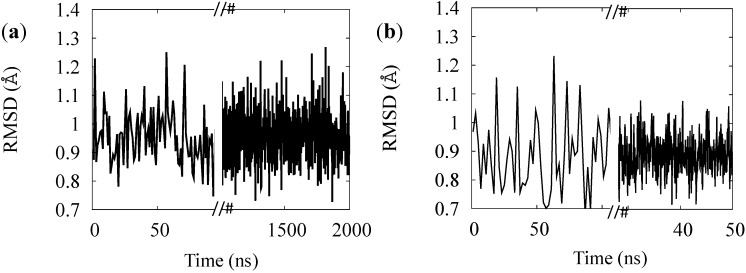
RMSD of the simulation of the BR (**a**) and for MR (**b**) rest states.

The rest states of BR and MR were used to test and finish the FF parameterization. For each of these cases we compared the B factor from RSMF evaluated on thermalized simulations at 100 K and at 300 K (red lines in Figure 3) with and without the constraining membrane potential (solid and dotted lines) with experimental B factors at 100 K (black line in panels (a) and (c) of Figure 3) or at 300 K from an atomistic simulation (for opsin only, panels (b) and (d) of Figure 3). The direct comparison of multi-scale simulation with experiment (red *vs*. black lines in panels (a) and (c)) indicates that the model gives systematically lower values especially in the helical regions. This effect is often observed and rather attributed to systematic errors in the experimental determinations [33]. In support to this, the experimental-simulation discrepancies are larger for the MR, for which the experimental data are less accurate. Thus, to solve this issue we performed *ad hoc* atomistic simulations of the completely hydrated proteins (opsins only, for simplicity) at 300 K and compared them with multi-scale simulations at the same temperature. The accuracy of the multi-scale model is considerably better in this case, and it is further improved by adding the containing potential of the membrane (solid *vs*. dotted lines in Figure 3). The stability of the models was also tested on the rest states (see Figure 4): we observe no instability up to the μsec time scales (for BR), in agreement with the fact that no relevant structural changes are observed in the proteins in absence of optical stimuli. 

**Figure 5:**
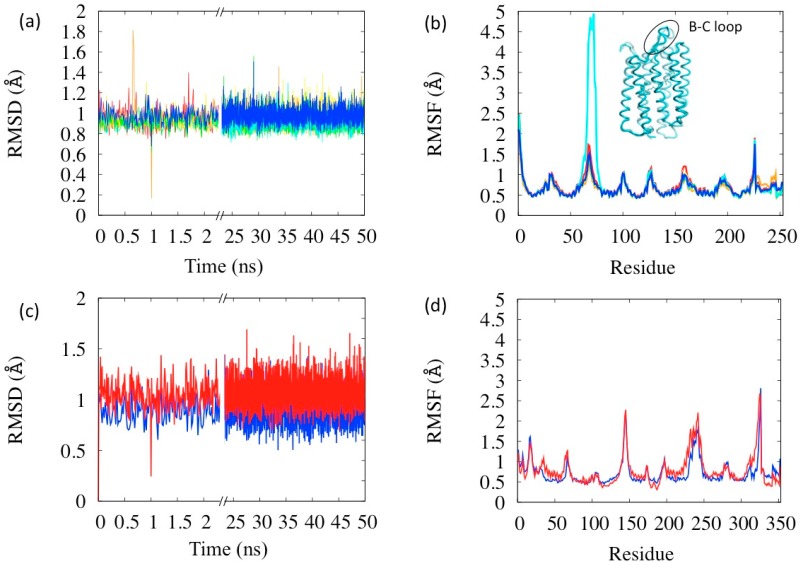
Simulations of the six BR photocycle states (**a**) RSMD and (**b**) RSMF. Color codes: Red = BR, orange = K, yellow = L, green = M, cyan = N, blue = O. The inset in (b) is a representative structure of the N state with B-C loop highlighted. Simulations of the two MR photocycle states (**c**) RMSD and (**d**) RMSF. Color codes: Blue = MR, red = LUMI.

With no parameter readjustment, the simulations were performed on all the states of the photocycle (complete analyses reported in the Supplementary Information, Section S6). The RSMD and RSMF evaluated in these simulations are reported in Figure 5a,b. All the simulations display similar characteristics to the BR state concerning stability: the average RSMD is in the range 0.92–0.99 Å, with small differences among the states. The analysis of the RSMF indicates that the more flexible areas are the loops, as expected. Interestingly the relative comparison between states indicates an anomaly in the state N (in cyan in the figure) whose B–C loop (res. 65–75) is the most mobile (see Figure 5b). This loop is rather a flap covering the extra-cellular opening of the helix bundle, which, in fact, has to open in the N state in order to let the proton passage. Thus its larger flexibility has a physiological meaning coherent with the protein function. Flap opening events are indeed visible during the simulation (inset in Figure 5b). The larger flexibility of the N state is probably also the reason why it was the one whose structure was latest resolved, because of difficulties in its crystallization [25]. Another interesting anomaly in fluctuations is in the retinal region (bead index 229–253 in Figure 5b), which are notably large in the state K (orange lines in Figure 5a,b). K state has, in fact, the retinal isomerized in the meta-stable state and its instability with respect to BR is estimated of about 10–15 kcal [34]. 

Simulations on the BR and LUMI states of bovine rhodopsin were also performed (Figure 5c,d) without changing the parameters set. Both states turn out to be stable and with RSMD within the common ranges, and as in the case of BR, the membrane mimicking potential has an important role in maintaining this stability. However, the LUMI state turns out to be little more flexible than the rest state (red *vs*. blue lines in Figure 5c,d), the helices being more mobile, in agreement with corresponding experimental B-factors.

### 3.2. Simulation of the Photo-Cycle Dynamics

**Figure 6:**
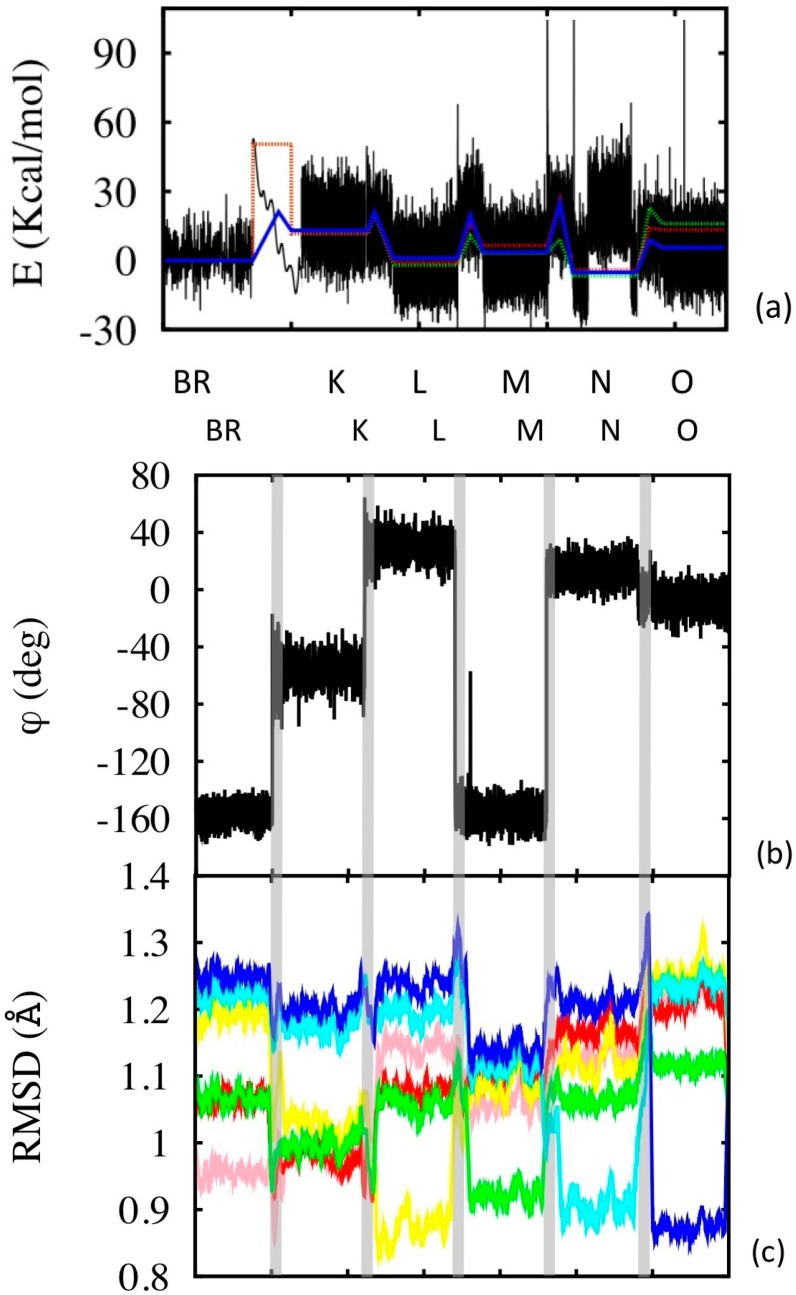
Photocycle simulations. (**a**) Configurational energy along the simulation (black line), and its average within the corresponding simulation segment (blue line). The simulation segments have different time lengths: the equilibrium state simulations are 50 ns long (2 μs in the case of BR, but only 50 ns are shown, see Supplementary Information for the complete simulation). The BR→K segment is 0.1 ps long, while the other transition segments are 10 ps long (the x axis scale is not proportional to the real simulation time, but enlarged according to visualization needs). Potential energies were aligned in pairs of subsequent states (or transition states) using common structures. The dotted lines are experimental values (green at pH = 7 and red at pH = 9). (**b**) Variation of the isomerization angle, evaluated using the four C atoms of the retinal enclosing the 13-bond. (**c**) RSMD evaluated along the simulation with respect to the different reference states (color code: pink with respect to BR, red to K, yellow to L, green to M, cyan to N and blue to O). The time scale of (b) and (c) does not correspond to that of (a), although the sequence of states is the same for the two (as indicated). The vertical grey bands separate the states and indicate the transitions.

The transition dynamics between states is simulated in each case applying the bi-stable FFs to the thermalized starting configuration, with the only exception of the BR→K transition. In this case, in fact, the transition does not proceed from the ground state, but from an electronically excited state, which brings the system in the “activated state” BR* from which the process is barrier-less. Thus in this case the FF of state K was directly applied to thermalized BR state, instead of the bi-stable one. The energies and some of the structural parameters are reported as a function of time in the different segments of simulation in Figure 6.

The BR→K passage occurs very fast. The isomerization occurs within the first 50 fsec, while the system relaxation takes a little longer, about 2 ps. These values are somewhat shorter than experimentally measured, in both cases by a factor nearly one half. This is a known effect of coarse graining: the kinetics is accelerated due to the elimination of degrees of freedom [18] and is observed also in the kinetics of the subsequent transitions. The fictitiously accelerated dynamics is also responsible for the spontaneous overcoming of the lower barriers in reasonable times (such as for instance the K→L in about 10 ps), while to see higher barrier overcome we forced the system towards the activated state by constraining the system along the reaction path (see the Supplementary Information, Section S5 for the reaction path definition) until the reaction occurs.

Figure 6a shows that the general energetic features are reproduced during potocycle. We remark that, while the relative energies of subsequent states are included into the parameterization, the barrier heights are not, and yet they are fairly well reproduced in the simulations. The “anomaly” of state N, namely the already mentioned opening of the B-C loop towards the cytoplasm is clearly visible in the corresponding segment of simulation, and produces a temporary increase of the configurational energy of the system. The plot of RSMD with respect to the six different reference states (Figure 6c) gives a quantitative idea of the structural variations and similarities between the states. In each case, obviously, the RSMD is low in the state taken as the reference. However some of the states (e.g., N, O) display the largest differences with respect to the others, while K, L and M are structurally not so dissimilar. 

### 3.3. Structural Features of the Transitions 

**Figure 7:**
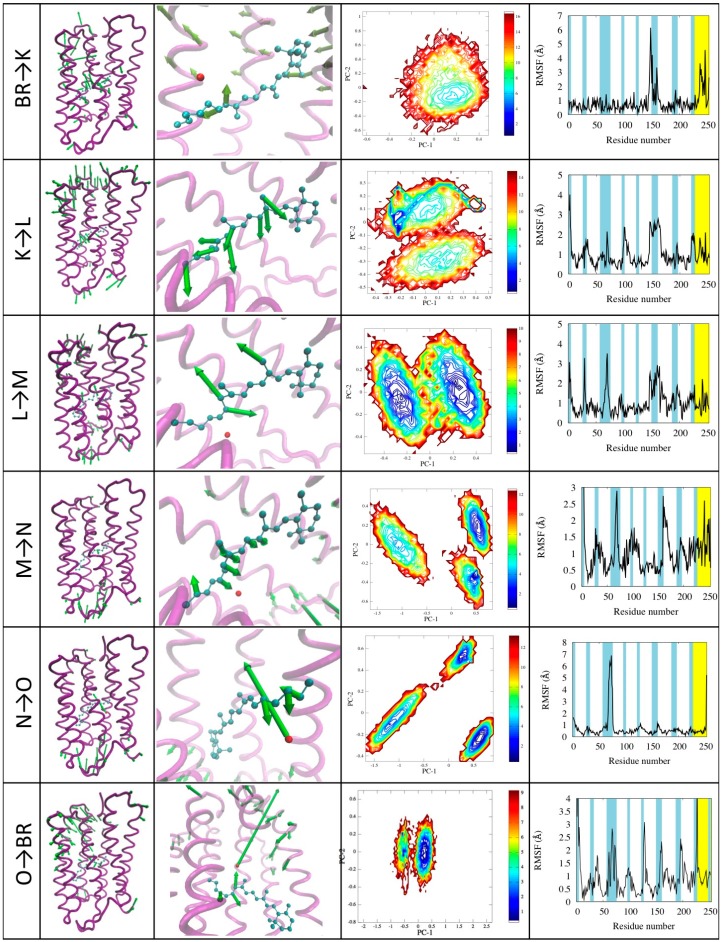
Illustration of the photocycle transitions. For each transition (indicated in the first column) the motion projected onto the first principal component is represented in the 3D structure with green arrows whose length is proportional to the displacement of the corresponding beads/atoms (second column; arrows shorter than a cutoff are not represented for clarity). A zoom on the active site shows in detail the motion of the retinal (third colum). The plots show the contour plots of the free energy landscape as a function of the first two principal components (fourth column) and the RSMF evaluated using the first principal component (fifth column).

We performed a detailed analysis of each transition trajectory with the aid of PCA analysis. The results are reported in Figure 7 and described below.

*BR→K.* This transition is dominated by the retinal isomerization, which occurs with small volume change due to a bicycle-like movement of the retinal (see Figure 7 first row, column 3). However, this is the triggering step that will produce the displacement of W402, which will later lose the hydrogen bonding to asp85. Other changes occur in the conformation of helix E. The first principal component describes the conformational change of the retinal, conveniently described by the 13-bond dihedral angle ϕ changing from the −156° to the −56° (see Figure 6c). The free energy landscape calculated on this transition shows that there is only one minimum corresponding to the K state. This is because, at variance with other simulations, this one is performed using a single state FF (that of state K) starting from the configuration of state BR.

*K→L.* During the transition, the retinal reaches a more distorted configuration, ϕ changing from −56° of the K state to +31° of the L state. This motion is found in the second and, to a lesser extent, in the fourth principal component, describing also the translation of the Schiff-base towards Asp85. This movement is related to the proton release [33]. The first principal component describes the fluctuation of the turn between helices E and F. The free energy landscape shows two distinct minima corresponding to the K and L states. A narrow blue area is visible in the L state, which is specifically related to the retinal motion within the active site.

*L→M.* In this transition ϕ changes from the +31° to −156°, corresponding to almost a complete rotation. The retinal conformation in M is very similar to the starting one, but the hydrogen bond network is different due to the breaking of the bond between the water molecule W402 and the Schiff base [23], which starts in our simulations in this phase, and is more clearly visible in the subsequent. The first principal components show the retinal motion and the displacement of turns at both sides of the protein. These movements also trigger the breaking of an hydrogen bond between Glu194 and Glu204 and the formation of a salt bridge between Glu204 and Arg82, followed by a large distortion in the extracellular half of helix 3 [35]. Also in this transition the free energy landscape clearly shows two distinct minima (see Figure 3c) corresponding to L and M states, and the paths connecting them. 

*M→N.* The second principal component shows motion of end of helix E and the turn between helices E and F on the cytoplasmic side (see Figure 7 forth row, column four). This corresponds to the creation of the opening in towards the cytoplasmic which becomes connected by a path to the center of the protein [25]. The ϕ dihedral goes back in the cis configuration passing from −156° to 25° of the N state. Noticeably, in this case the free energy landscape shows three different minima: Two of them are M and N states as expected, while the third one is a metastable intermediate [25]. This differs from the M and N states mainly for changes in the retinal and in the Schiff base linkage: in the M state the Schiff base is capable of exchanging protons with asp85, while in the intermediate the protonation is dependent on asp96 [24]. We remark that this state was not explicitly included in the model, yet spontaneously emerges from the simulations.

*N→O.* This transition is characterized by the motion of helix C shifting towards helix E, closing the proton release channel and ejecting the water molecule W402. These motions emerge from the analysis of the first and second principal component (see Figure 7 with “fifth row, column four”). This mechanism is similar to a valve controlling the mono-directionality of the proton transport [26]. In the second principal component we also see small movements of the retinal. As in the previous case, besides the two N and O states a third one emerges characterized by small structural differences mainly in helices C and D and in the E-F turn, which also little affect the conformations of helices F and G, in agreement with what found in the literature [25]. Again we remark that this state spontaneously emerge from the model, not explicitly included in it.

*O→BR.* The return to the ground state is characterized by the rearrangement of the whole structure and in particular the turn regions. The retinal returns in the *all-trans* conformation rebuilding the hydrogen bond network that stabilizes the active site. The first principal component describes turns motion on both sides of the protein, the retinal movement and movements involving helices near the proton release channel. No metastable transition states are detected in this case. A movie of the complete photo-cycle is provided in the Supplementary Information.

## 4. Experimental Section 

### 4.1. Simulation Protocols and Tools

The classical molecular dynamics simulations were performed either within the microcanonical ensemble using Verlet algorithm [36] for the dynamics or canonical ensemble, using the Berendsen thermostat [37], with coupling constant τ = 1 fs for the ground state simulation and τ = 50 fs for other states. This change does not affect the model stability. The timestep for integration was 1 fs, which provides a sufficient integration both for all the interactions. 

Annealing procedures were applied to equilibrate the systems, involving a starting minimization and a subsequent slow eating in steps 10, 20, 50, 100, 200 and 300 K during a total of 6 ns (1 ns each heating step). The production runs were at least 50 ns long. 

Simulations were performed with the DL_POLY_2.20 package [38]. The input files for atomistic simulations inputs for OPLS FF were created with the DL_FIELD package [39], while all the inputs for multi-scale models were created with in-house build software (available upon request). Double well functional forms needed for the multi-stable models are not implemented in the standard version of DL_POLY. Thus we implemented them in additional modules for DL_POLY, which are available upon request. Atomistic simulations were also performed with the OPLS-AA force field [40] (using the GROMACS package [41]) of the completely hydrated native state of BR. In this case a cubic box of 75 Å [42] side was used with periodic boundary conditions. 

### 4.2. Simulation Analysis

The root mean squared deviation from reference structures and root mean squared fluctuations were evaluated with the standard formulas:

$$RMSD(t)=\sqrt{\frac{1}{n}\overset{N}{\underset{i=1}{\sum }}{\left(r_{i}\left(t\right)-r_{i}\left(0\right)\right)}^{2}}$$

where *N* is the number of atoms in the simulation, ***r****_i_*(*t*) is the position vector of atom *i* at time *t*, and ***r*^0^***_i_* the reference configuration (usually the starting one), and:

$$RMSF_{i}=\sqrt{\frac{1}{T}\overset{T}{\underset{t_{j}=1}{\sum }}{\left(r_{i}\left(t_{j}\right)-\langle r_{i}\rangle \right)}^{2}}$$

with $\langle r_{i}\rangle$ the time averaged position of atom *i*. The RSMF is then compared with the root mean squared displacement evaluated from the experimental B factor by means of:

$$B_{i}=8\pi^{2}\langle \Delta {r_{i}}^{2}\rangle$$

The principal component analysis (PCA) [43,44,45,46] of the trajectories is used to evaluate the essential dynamics in simulations. To this aim the covariance matrix:

$$\sigma_{ij}^{\alpha \beta }=\langle (r_{i}^{\alpha }-\langle r_{i}^{\alpha }\rangle )\left(r_{j}^{\beta }-\langle r_{j}^{\beta }\rangle \right)\rangle$$

is evaluated, diagonalised and analysed by means of the ProDy [47], plugin of the visualization software VMD [48] (here the Greek letters are Cartesian indices). The trajectories are then projected onto the first ten eigen-modes ordered by eigenvector (*i.e.*, the principal components), which represents the essential dynamics of the systems. VMD is also used to create all graphical representations of the proteins. 

## 5. Conclusions and Perspectives 

In this work we reproduced the complete photocycle of the bacteriorhodopsin using a multi-scale and multi-stable model. The resolution is the minimal necessary to reproduce all the structural characteristics, namely, quasi-atomistic in the retinal active site, and one-bead per amino-acid for the opsin. Thanks to this very simple representation and to the completely classical treatment, macroscopic scales can be reached in the simulations, the photocycle dynamics was addressed with very modest computational resources (single processor). 

Thanks to physics based (top-down) parameterization including the experimental energy differences between the states interpolating FFs between the states are obtained, which reproduce not only relative free energies but also the barriers, whose numerical value was not explicitly included, yet spontaneously emerge as an effect of the accurate parameterization of the transition Force Fields. This is also true for the first BR*→K, transition, occurring in the excited state, indicating that also the QM level of the description is somehow included though not explicitly.

The structural features of all the states are also well reproduced, as an effect of the accurate parameterization and of the flexibility of the hybrid UA/CG model. The kinetics of the barrier overcoming is somewhat accelerated, which is a well-known effect of the reduction of the resolution, although in the order of magnitude of the relevant steps are reproduced. The detailed PC analysis highlights a number dynamical features of the transitions, which are compatible with the current knowledge on the BR photocycle and help the interpretation of the different phases of the cycle on a structural basis.

A certain amount of *a priori* knowledge is included in the model, namely the (low resolution) structural knowledge of the six reference states of the bacterial rhodopsin. However, we observe that this knowledge is used to include only a partial bias into the model, which, in fact, is very flexible and able to reproduce states and dynamics far from those. Consequently, and noticeably, metastable transition state not explicitly included in the parameterization, spontaneously emerge from the simulations. These are quantitatively comparable with data available in the literature. This constitutes an independent validation of the model and of the procedure adopted to build it.

The model is used with no re-parameterization to simulate the known states of the bovine rhodopsin. The equilibrium features are well reproduced, and as far as the experimental data allow comparison, the agreement is quantitative. This was not obvious, given the non-negligible structural difference between the eukaryotic and prokaryotic rhodopsins. In conclusion, this model has proven accurate and robust to simulate equilibrium states of the helical transmembrane proteins and their transitions. In addition, given the simplicity of the model, we believe it is amenable to be used to simulate larger systems including rhodopsins. Immediate applications which do not need a reparameterization of the model include the study of the multimeric organization of the bacterial rhodopsin, or the G-protein-rhodopsin complex. Their dynamics could be addressed on the macroscopic time scales, in simulations, in spite of their size, due to the extreme simplification of the model.

## Supplementary Materials

Supplementary materials can be accessed at: http://www.mdpi.com/1420-3049/19/9/14961/s1.

## Supplementary Files

Supplementary File 1
