# Supplementary material for: A Multi-Scale–Multi-Stable Model for the Rhodopsin Photocycle
Source: Molecules. 2014 Sep 18;19(9):14961–78. doi: 10.3390/molecules190914961 (PMC6271392; doi:10.3390/molecules190914961)
Supplement: Supplementary File 1 [file molecules-19-14961-s001.pdf]

Supplementary Materials

S1. Phenomenology of the Photo-Cycles

Figure S1 and Table S1 report the known phenomenology about the BR and MR photocycles. For each state and transition, the absorption wavelengths and transition times are reported. For the first transition the two different times refers to the chromophore isomerization and subsequent relaxation respectively. For BR also the free energies are reported in the table (referred to the BR rest state, the first in the table), measured in different pH conditions. The plots report correlation between these quantities. Figure S1a shows a clear correlation between the barrier height (measured as difference beteween the activated state and the starting one, green and red dots = experimental data in different conditions, see caption) and the transition time, as expected. This correlation is reproduced in simulations (black dots).

Figure S1. (**a**) Correlation plots between absorption wavelength and state life time for bacterial rhodopsin (black) and bovine rhodopsin (grey). (**b**) correlation plot between transition barriers height and transition time for bacterial rhodopsin. Free energies are evaluated at pH 7 (green) and 9 (red), while the black dots are from the simulations. Data for energies, times and wavelengths are numerically reported in Table S1 with their references. Times are in log scale.

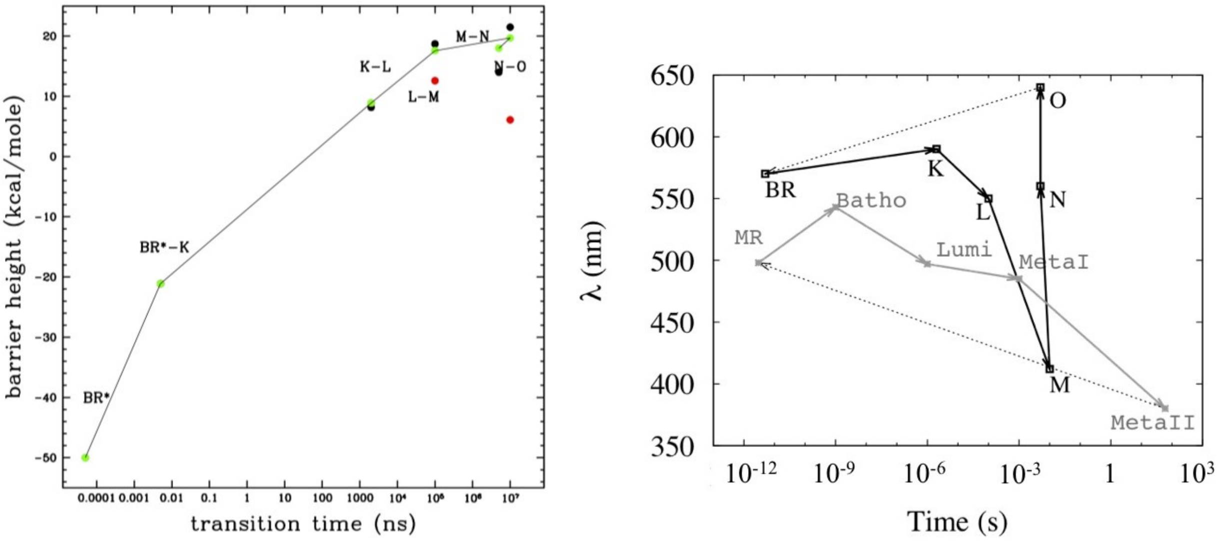

(**a**) (**b**)

Conversely, the correlation between the transition wavelengths and the transition times (Figure S1b) does not show a clear trend. The optical properties are, in fact, more related to the state of the retinal (*cis-trans*), of the hydrogen bond network surrounding it and the protonation state of the Schiff base [1–5].

Table S1. Adsorption wave-length, the transition times, and relative energies of each state of the photo-cycle of BR (upper table) and of MR (lower table. Data from [6–10]).

BR						
**Id**	**λ_{ass} [nm]**	**Transition Time**	**E (kcal/mole) pH = 5**	**E (kcal/mole) pH = 7**	**E (kcal/mole) pH = 9**	**E (kcal/mole) (sim)**
BR	570		0	0	0	0
BR*→K		100fs–5ps	50.5	50.5	50.5	50.50
K	590		11.7	11.7	11.7	11.7
K→L		2μs	20.7	20.6	20.6	
L	550		−0.3	−2	−0.9	5.5
L→M		100μs	17.4	10.6	16.7	
M	412		1.7	3.2	6.5	1.25
M→N		10ms	18.1	9.3	26.2	
N	560		34.4	−6.7	−4	0.08
N→O		5ms	2.5	22.8	14	
O	640		16.3	16	13.3	2.75
O→BR		5ms				

MR		
**Id**	**λ_{ass} [nm]**	**Transition Time**
RHO	498	
RHO*→BATHO		~15–50ps
BATHO	543	
BATHO→LUMI		1ns
LUMI	497	
LUMI→METAI		1μs
METAI	485	
METAI→METAII		1ms
META II	380	
METAII→OPSIN		1min
OPSIN		

S2. Reference States Choice

The reference structures for the photo cycle states were chosen as follows. For each optical state, we searched in the PDB [11,12] repository the available structures. Among these, we chose the best compromise between resolution and completeness. When possible, we also excluded the mutants and choose crystallization conditions as far as possible natural in terms of temperature and illumination. We also needed the same number of residues for each of the cycle photo-state. Thus when necessary the structures were completed using data from other structures of the same state. The structures used and some of their features (PDB code, reference, publication year) are reported in Table S2, and represented in Figure S2.

Table S2. List of data used to build the reference structures for the photocycle states. The PDB codes are reported (in case a second structure is used to complete the first, the list of the AA used is also reported), the resolution, and the publication year of the structure.

	State	PDB ID Structure	Resolution (Å)	Publ. Year
BR	BR	1M0K(1) + 1FBB [153–166]	1.43	2002
	K	1M0K(2) + 1IXF [152–168]	1.43	2002
	L	1O0A(1) + 1UCQ [150–168]	1.62	2003
	M	1M0M(1) + 2ZZL [150–173]	1.43	2002
	N	1P8U + 1M0M [151–163]	1.62	2003
	O	3VI0	2.30	2012
MR	MR	1U19	2.2	2004
	LUMI	1F88	2.8	2000

Figure S2. (**a**) Superimposed representative structures of the multi-scale model of BR. (**b**) zoom of the active site (UA representation). Color code: Red = BR, orange = K, yellow = L, green = M, cyan = N, blue = O. (**c**) superimposed representative structure of MR and LUMI (**d**) Zoom of the active site. Color code: red = MR, blue = LUMI. In the retinals, the isomerizing bonds are highlighted.

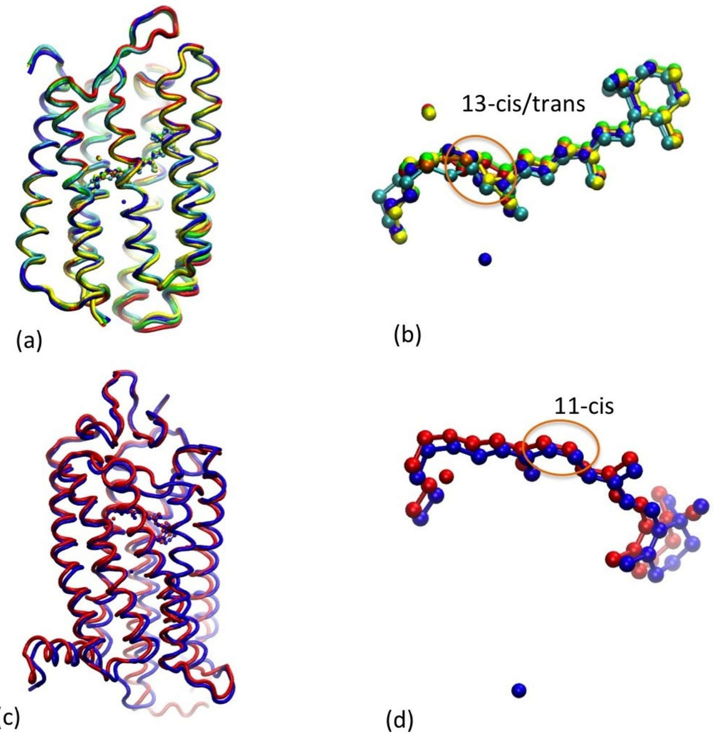

S3. Force Field Details and Parameters

The explicit form of each of the FF term is the following:

$$U_l = \sum_i^n u_l(r_i) \qquad U_\theta = \sum_i^n u_\theta(\theta_i) \qquad U_\Phi = \sum_i^n u_\Phi(\Phi_i)$$

$$U_{loc} = \sum_{r_{ij}<r_{cut}}^{n} u_l(r_{ij}) \qquad U_{non-loc} = \sum_{r_{ij}>r_{cut}}^{n} u_{nl}(r_{ij})$$

The functional forms used for the *u*s depend on the specific bead type and class of interaction, as specified in Tables S3 and S4. The functional forms are chosen among the following:

Constraint = holonomic constraint (maintained by the SHAKE algorithm [13,14])
harmonic = $\frac{1}{2}k_r(r(t) - r_0)^2$
harmonic cosine = $\frac{1}{2}k_\theta\left(cos(\theta(t)) - cos(\theta_0)\right)^2$
cosine = $A[1 + cos(m\phi - \delta)]$
Morse = $\varepsilon\left[\left\{1 - e^{-\alpha(r(t)-r_0)}\right\}^2 - 1\right]$
12–6 = $\left(\frac{a}{r(t)^{12}}\right) - \left(\frac{b}{r(t)^{6}}\right)$

Table S3. Description of resolution separation in the multi-scale model, and other features of the model. The bead resolution level (CG = one bead per amino-acid resolution, UA united atom) and type (different type have generally different parameterization, as specified below) is also reported. The tethered beads (to simulate the membrane effect) are listed by their subsequent amino-acid number.

	**Bead Resolution**	**Bead Type**	**Location**
	**Bacterial Rhodopsin**		
Cα from residue 1 to 227	CG	Ca	Opsin
Retinal Carbon	UA	Cr	
Shiff base nitrogen	UA	Ns	**Active Site**
Lys-216 side-chain heavy atoms	UA	Ly	
Water Oxigen	UA	Ow	
List of tethered beads:	11, 15, 18, 22, 25, 44, 51, 52, 55, 59, 81, 84, 88, 91, 92, 95, 99, 109, 110, 113, 114, 116, 117, 120, 121, 139,140, 142, 143, 146, 147, 150, 151, 172, 173, 176, 177, 180, 183, 184, 187, 188, 206, 207, 210, 213, 214, 218, 221, 222.		
	**Mammalian (Bovine) Rhodopsin**		
Cα from residue 1 to 326	CG	Ca	
Retinal Carbon	UA	Cr	
Shiff base nitrogen	UA	Ns	**Active Site**
Lys-296 side-chain heavy atoms	UA	Ly	
Water Oxigen	UA	Ow	
List of tethered beads:	35, 38, 39, 42, 43, 45, 46, 49, 50, 53, 54, 57, 60, 63, 64, 74, 81, 85, 88, 89, 92, 93, 96, 112, 116, 151, 154, 155, 158, 159, 162, 165, 166, 169, 170, 202, 205, 206, 209, 210, 213, 214, 217, 220, 221, 224, 225, 227, 228, 249, 252, 256, 259, 260, 263, 266, 267, 270, 271, 274, 290, 291, 294, 297, 304, 305, 308, 314, 317, 321.		

The optimized parameters and functional forms of the multi-scale model for single states are reported in Table S4.

Table S4. FF parameters of the multi-scale model for BR. The distances (r_0 r_{cut}) are in Å, the angles (θ, ϕ) in deg, the costants k_θ, K_{teth} and A in Kcal/mol and for the 12–6 potential a and b are in $Å^{12}$ and $Å^6$ respectively. The value of the r_{cut} for the separation between local and non local part of the non bonded interactions is uniformly set at 8.5 Å.

FF Term	Analytical Form	Parameters
	Opsin	
U_l	constraint	
U_θ	harmonic cosine	$k_\theta = \begin{cases} 110 & \theta < 97 \\ 20 & 97 < \theta < 115 \\ 70 & 115 < \theta < 130 \\ 90 & 130 < \theta < 150 \\ 10 & \theta > 150 \end{cases}$
U_ϕ	cosine	$A = \begin{cases} 25 & \phi < 80 \\ 5 & \phi > 80 \end{cases}$
U_{local}	Morse	$\varepsilon = 3.9e^{-\left(\frac{r}{6.15}\right)^6} + 0.05$ $\alpha = 2e^{-\left(\frac{r}{6.2}\right)^8} + 0.7$
$U_{non-local}$ [15]	Morse	$\varepsilon = 0.01$ $\alpha = 0.7$ $r_0 = 9.5$
U_{teth}	harmonic	$K_{teth} = 2$
	Active site [16]	
U_l	constraint	
U_θ	harmonic	$k_\theta = 63$
U_ϕ	cosine	$A = 2$
U_{local}	morse	$\varepsilon = 0.12$ $\alpha = 2e^{-\left(\frac{r}{6.2}\right)^8} + 0.7$
$U_{non-local}$		
$Cr - Cr$	12–6	$a = 538$ $b = 11.9$
$Cr - Wo$		$a = 1285145$ $b = 673.2$
$Cr - Ly$		$a = 372.2$ $b = 8.6$
$Wo - Wo$		$a = 142.5$ $b = 9.2$
$Wo - Ly$		$a = 712422$ $b = 494$
$Ly - Ly$		$a = 205$ $b = 6.4$
$Ns - Ly$		$a = 205$ $b = 6.4$
$Ns - Wo$		$a = 142.5$ $b = 9.2$
$Ns - Cr$		$a = 372.2$ $b = 8.6$
$Ns - Ns$		$a = 205$ $b = 6.4$
	AA-UA interface	
U_{bonds}	constraint	
U_{local}	Morse	$\varepsilon o\left(8e^{-\left(\frac{r}{6.3}\right)^4} + 0.05\right) * 0.05$ $\alpha 0.05e^{-\left(\frac{r}{6.3}\right)^4} + 0.7071$
$U_{non-local}$ [16]		
$Ca - Cr$	Morse	ε2‑6e − α2‑6e − 17 $r_0 = 6.5$
$Ca - Wo$	12–6	$a = 695928$ $b = 560$
$Ca - Ly$	12–6	$a = 815008$ $b = 461$
$Ca - Ns$	12–6	$a = 815008$ $b = 461$
$U_{h-bonds}$	Morse	$\begin{cases} \varepsilon = 1.9 \quad \alpha = 2 & donor \\ \varepsilon = 5.0 \quad \alpha = 1.85 & acceptor \end{cases}$

S4. Interface Parameters Optimization Procedure

Interface interactions between the active site and the rest of the protein need a *de novo* parameterization. These interactions involve U_l, U_θ, U_Φ of the retinal-lys side chain linkage, a number of non-bonded U_{loc} interactions between the retinal and residues facing it. The bonding potentials are parameterized combining the partially biased model and the UA model: the structural parameters (equilibrium distances, angles and dihedrals) are taken from the reference structures, while elastic constants are obtained by the UA parameters.

The U_{loc} term is represented as a sum of Morse potentials (see previous section). The parameters of this term are fitted on energy profiles evaluated by atomistic simulations using the OPLS FF, as a function of the independent variables, by constraining it to sampled values and optimizing the systems with respect to all the other coordinates. As an example, Figure S2 illustrates the procedure to evaluate the Morse term representing the hydrogen bond between the water and an aspartic acid and between two water molecules. In this case the independent variable is the distance between the beads representing oxygen and the aspartic acid (Cα of asp). As it can be seen from the Figure S3, the Morse potential fits very well the atomistic energy profile. The obtained value for the width parameter α is little less than 2 $Å^{-1}$, larger than the other local interactions (see Table S2) as an effect of the deeper and shorter range hydrogen bonding interactions, with respect to other local ones. The value obtained for ε is further optimized. In fact, as an effect of the resolution reduction and of the topology of the local interaction, in our model each hydrogen bond is described by a sum of several Morse interaction. This implies a scale factor to be applied to ε, which was estimated to 0.005 by comparing the multi-scale model with atomistic simulations in the interface region.

Figure S3. Fitting procedure to parameterize the hydrogen bonding CG-UA or UA-UA interactions (**a**,**c**): atomistic representations of the elements involved in the hydrogen bond (water and aspartic acid in (a) and two waters in (c)). (**b**) energy profile evaluated with the OPLS FF, as a function of the distance between beads (red dots) and its fit with a morse potential (black line).

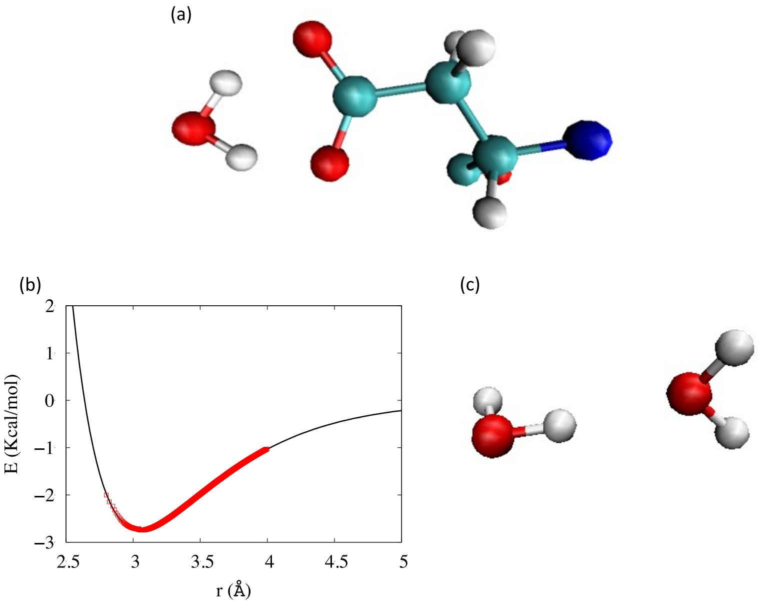

The hydrogen bonds formed by water molecule W402, namely with Asp85, Asp212 and Lys216 where treated explicitly and individually, thus in this case no rescaling is needed. In these bonds, water acts as a donor or as acceptor [5]. We added other three potentials to the U_{loc} term, $U_{h\text{-}bond}$, described by a Morse potential with ε and α depending on the type of hydrogen bond created according to the procedure described above. In particular if the water molecule acts as a donor, the binding is higher, see Table S2. The final values for the parameters are reported in Table S2.

S5. Multi-Stable Models Building

As explained in the main text, except for the first transition, the multi-stable models are obtained by combining in couple subsequent models for the single states. The model is built in order to reproduce the relative energies of the states taken from experiment, δ. The bi-stable models are composed of the sum of a number of double well potentials, thus the amount of δ must be distributed by these. Table S5 describes how this distribution is performed. First, the number of term treated with the double well form in place of the single well one is limited to those whose equilibrium values difference overcomes a threshold (fixed at 10% for distance depending interactions and to 5% for angles and dihedrals), while a single well is used otherwise. This limits the use of double well potentials to the terms which substantially contributes to the difference of the structure. This criterion is applied to bonding terms and to local non-bonded. It is to be noted that if a given couple i–j is present only in one of the two states, then it is represented with a double well Morse where the second well has its minimum corresponding to the value of the generic non local-non bonded potentials. Then the value of δ is distributed among those double well interactions which are mainly involved in the transition, which are reported in Table S5, namely those included in the active site. The physical meaning of this procedure is that the energy differences between the different states are mainly due to conformational diversity arising from the active site.

Table S5. List number and type of interactions treated with the double well form for each transition. Those in the active site are explicitly listed. The value of δ (reported) is equally distributed.

Transition	Bonds	Angles	Dihedrals	Dihedrals in the Active Site	δ (Kcal/mol)
K-L	133	212	193	Cr12 − Cr13 = Cr14 − Cr15	12.0
L-M	155	225	191	Cr12 − Cr13 = Cr14 − Cr15	10.0
				Cr14 − Cr15 = Ns − Ly	10.0
M-N	280	223	205	Cr12 − Cr13 = Cr14 − Cr15	1.2
N-O	335	244	225	Cr12 − Cr13 = Cr14 − Cr15	2.0
				Cr14 − Cr15 = Ns − Ly	2.0
				Cr15 = Ns – Ly = Ly	1.2

S6. Details of Single States Simulations

Figure S4 reports the energy variation of *vs.* time during the single state dynamics simulations. The rest state simulation is 2 μs long and the other states are 50 ns long. As noted in the main text, the N state simulation shows a change in conformation of the turn between helix E and F, an "opening",

which results in a change in energy in the dihedral term of 15 Kcal/moL. The duration of the event is 33 ns. All other states do not show noticeable conformational changes during dynamics.

Figure S4. Energies and temperature *vs.* simulation time for each single state simulation of BR (state indicated). (**a**) Blue = bond angles energy, dihedrals energy; (**b**) temperature; (**c**) total potential energy; (**d**) black $U_{non\text{-}loc}$, red U_{loc}.

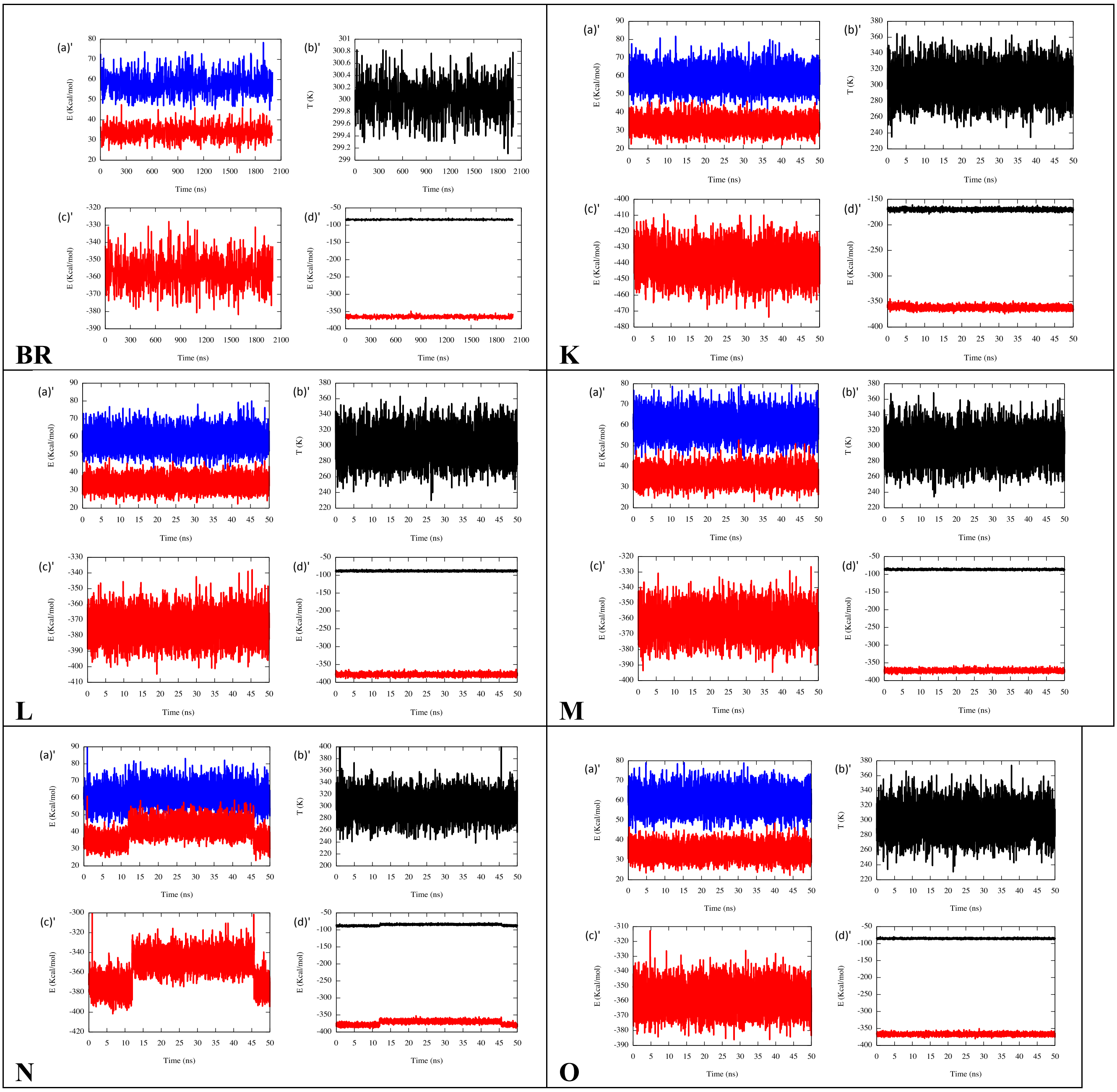

S7. Details of the State Transitions

For the analysis of the different state transitions it is useful to define a reasonable “reaction path” for each of them. For the BR-K and L-M transitions, this can be recognized to be the dihedral angle around the 13th bond in the retinal chain, namely that describing the bond isomerization. For each transition, we determined a series of configurations at fixed reaction coordinates letting all the others

to relax. We remark that these configurations approximately interpolate between the starting and final states, and are used only for rough evaluation of the energetics during the transition and test in a simple way the effectiveness of the double well approach. The energy of the intermediate configuration was evaluated in each case with three FFs: that of the starting state, that of the final state and the double well FF connecting the two states. Sample results of energy profiles are reported in Figure S5.

The total potential energies (red) and temperatures (black) along the transition simulations are reported in Figure S6. As noted in the main text, small or null barrier transitions (e.g. K-L or BR*-K) occur fast or spontaneously. Other transitions are induced by forcing the system along the above determined paths (forcing the coordinate to increase smoothly), up to the top or the barrier. The energy profiles are then re-evaluated along the simulations and reported in the main text. During all transitions, the temperature is maintained constant at 300K with the Berendsen thermostat (time constant 1 fs).

Figure S5. Energy profiles *vs.* the reaction coordinate for selected BR photo-cycle transtions. In green, the energy calculated with the first state FF, in red with the second state FF, in black the energy calculated with the double well FF. In panels (**a**) the total energy E_{tot}, (**b**) the bond angle energy, in (**c**) the dihedrals energy, (**d**) the non-local -non bonded energy, (**e**) the local non bonded, with solid lines (scale on the left), and the non-local energy, with dashed lines (scale on the right).

BR-K

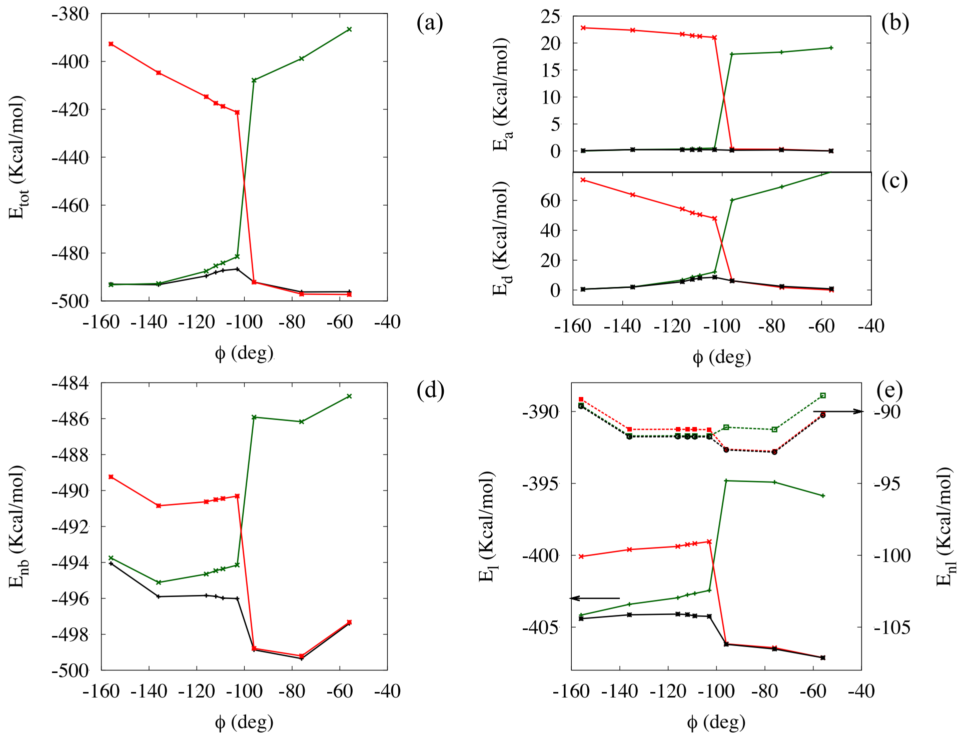

Figure S5. *Cont.*

L-M

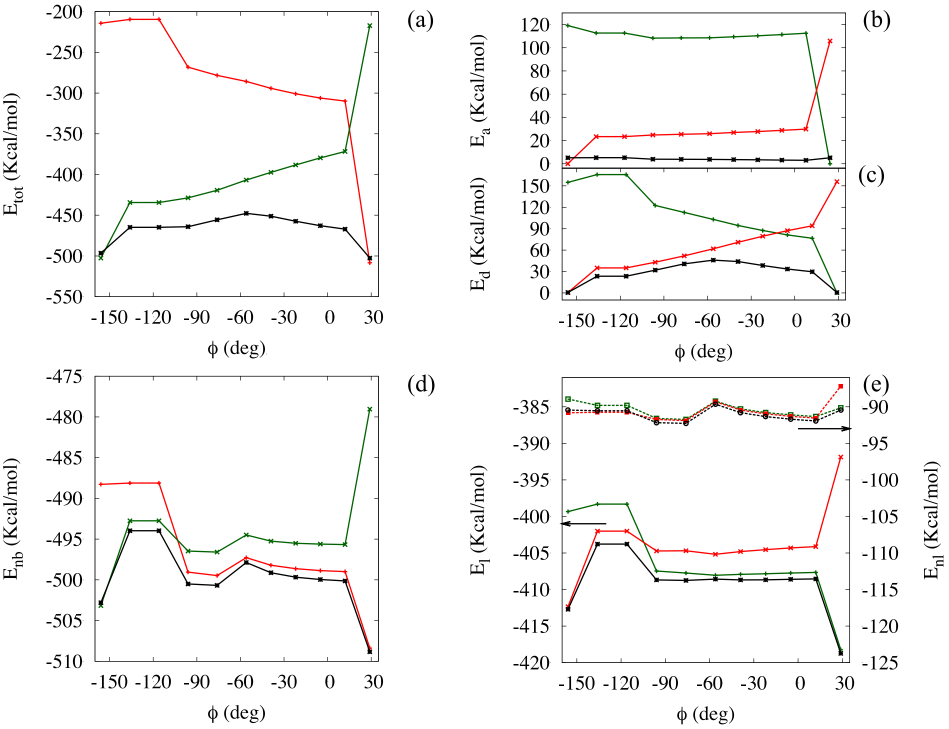

Figure S6. potential energy, in red (scale on the right in kcal/mole), and temperature, in black (scale on the left, in K), for each photo-cycle transition: (**a**) BR* to K, (**b**) K to L, (**c**) L to M, (**d**) M to N and (**e**) N to O.

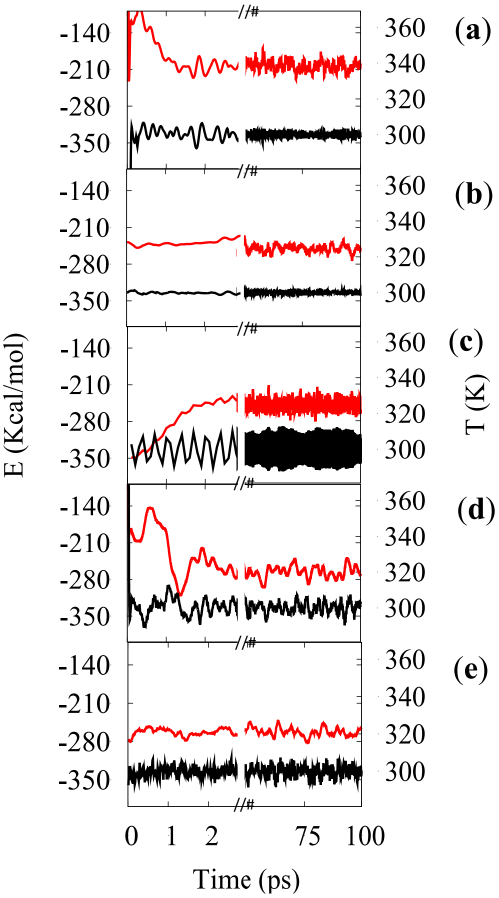

References

1. Schobert, B.; Cupp-vickery, J.; Hornak, V.; Smith, S.O.; Lanyi, J.K. Crystallographic Structure of the K Intermediate of Bacteriorhodopsin: Conservation of Free Energy after Photoisomerization of the Retinal. *J. Mol. Biol.* **2002**, *2836*, 715–726.
2. Edman, K.; Nollert, P.; Royant, A.; Belrhali, H.; Pebay-Peyroula, E.; Hajdu, J.; Neutze, R.; Landau, E.M. High-resolution X-ray structure of an early intermediate in the bacteriorhodopsin photocycle. *Nature* **1999**, *401*, 822–826.
3. Lanyi, J.K.; Schobert, B. Crystallographic Structure of the Retinal and the Protein after Deprotonation of the Schiff Base: The Switch in the Bacteriorhodopsin Photocycle. *J. Mol. Biol.* **2002**, *2836*, 727–737.
4. Chen, D.; Lanyi, J.K. Structural changes in the N and N' states of the bacteriorhodopsin photocycle. *Biophys. J.* **2009**, *96*, 2779–2788.
5. Zhang, J.; Yamazaki, Y.; Hikake, M.; Murakami, M.; Ihara, K.; Kouyama, T. Crystal structure of the O intermediate of the Leu93→Ala mutant of bacteriorhodopsin. *Proteins* **2012**, *80*, 2384–2396.
6. Lozier, R.H.; Xie, A.; Hofrichter, J.; Clore, G.M. Reversible steps in the bacteriorhodopsin photocycle. *Proc. Natl. Acad. Sci. USA* **1992**, *89*, 3610–3614.
7. Hermone, A.; Kuczera, K. Free-Energy Simulations of the Retinal Cis→Trans Isomerization in Bacteriorhodopsin. *Biochemistry* **2010**, *2960*, 2843–2853.
8. Birge, R.R.; Cooper, T.M. Energy storage in the primary step of the photocycle of bacteriorhodopsin. *Biophys. J.* **1983**, *42*, 61–69.
9. Ludmann, K.; Gergely, C.; Váró, G. Kinetic and thermodynamic study of the bacteriorhodopsin photocycle over a wide pH range. *Biophys. J.* **1998**, *75*, 3110–3119.
10. Onufriev, A.; Smondyrev, A.; Bashford, D. Proton Affinity Changes Driving Unidirectional Proton Transport in the Bacteriorhodopsin Photocycle. *J. Mol. Biol.* **2003**, *332*, 1183–1193.
11. Berman, H.M.; Westbrook, J.; Feng, Z.; Gilliland, G.; Bhat, T.N.; Weissig, H.; Shindyalov, I.N.; Bourne, P.E. The Protein Data Bank. *Nucleic Acids Res.* **2000**, *28*, 235–242.
12. Wickstrand, C.; Dods, R.; Royant, A.; Neutze, R. Bacteriorhodopsin: Would the real structural intermediates please stand up? *Biochim. Biophys. Acta* **2014**, doi: 10.1016/j.bbagen.2014.05.021.
13. Smith, W.; Forester, T.R.; Todorov, I.T. *The DL_POLY Classic User Manual*, Version 1.0; STFC Daresbury Laboratory Daresbury: Warrington WA4 4AD Cheshire, UK, 2010.
14. Smith, W.; Forester, T.R. DL_POLY_2.0: A general-purpose parallel molecular dynamics simulation package. *J. Mol. Graph.* **1996**, *7855*, 136–141.
15. Tozzini, V.; Mccammon, J.A. A coarse grained model for the dynamics of flap opening in HIV-1 protease. *Chem. Phys. Lett.* **2005**, *413*, 123–128.
16. Weiner, S.J.; Kollman, P.A.; Case, D.A.; Singh, U.C.; Ghio, C.; Alagona, G.; Profeta, S.; Weiner, P.A. New Force Field for Molecular Mechanical Simulation of Nucleic Acids and Proteins. *J. Am. Chem. Soc.* **1984**, *106*, 765–784.
